# Association between antenatal corticosteroids and neonatal outcomes among very preterm infants born to mothers with hypertensive disorders of pregnancy: a multicenter cohort study

**DOI:** 10.1186/s13052-025-01909-9

**Published:** 2025-03-13

**Authors:** Mengya Sun, Aimin Qian, Xianghong Li, Ruimiao Bai, Ping Cheng, Xinyue Gu, Yanchen Wang, Yun Cao, Wenhao Zhou, Shoo K. Lee, Hong Jiang, Siyuan Jiang, on behalf of the Chinese Neonatal Network (CHNN), Lin Yuan, Tongling Yang, Hao Yuan, Li Wang, Yulan Lu, Chao Chen, Lizhong Du, Xiuyong Chen, Huayan Zhang, Xiuying Tian, Jingyun Shi, Zhankui Li, Chuanzhong Yang, Ling Liu, Zuming Yang, Jianhua Fu, Yong Ji, Dongmei Chen, Changyi YANG, Rui Chen, Xiaoming Peng, Ruobing Shan, Shuping Han, Hui Wu, Lili WANG, Qiufen Wei, Mingxia Li, Yiheng Dai, Wenqing Kang, Xiaohui Gong, Xiaoyun Zhong, Yuan Shi, Shanyu Jiang, Bing Sun, Long Li, Zhenlang Lin, Jiangqin Liu, Jiahua PAN, Hongping Xia, Xiaoying Li, Falin Xu, Yinping Qiu, Li Ma, Ling Yang, Xiaori He, Yanhong Li, Deyi Zhuang, Qin Zhang, Wenbin Dong, Jianhua Sun, Kun Liang, Huaiyan Wang, Jinxing Feng, Liping Chen, Xinzhu Lin, Chunming Jiang, Chuan Nie, Linkong Zeng, Mingyan Hei, Hongdan Zhu, Hongying MI, Zhaoqing Yin, Hongxia Song, Hongyun Wang, Dong Li, Yan Gao, Yajuan Wang, Liying Dai, Liyan ZHANG, Yangfang Li, Qianshen Zhang, Guofang Ding, Jimei Wang, Xiaoxia Chen, Zhen Wang, Zheng Tang, Xiaolu Ma, Xiaomei Zhang, Xiaolan Zhang, Fang Wu, Yanxiang Chen, Ying Wu, Joseph Ting

**Affiliations:** 1 Department of Neonatology, The Affiliated Hospital of Qingdao University, 16 Jiangsu Road, Qingdao, Shandong 266000 China; 2 Department of Neonatology, Children’S Hospital of Nanjing Medical University, Nanjing, Jiangsu 210008 China; 3 Department of Neonatology, Northwest Women’S and Children’S Hospital, Xi’an, Shanxi, 710061 China; 4 Department of Neonatology, Children’S Hospital Affiliated to Zhengzhou University, Zhengzhou, Henan 450018 China; 5 NHC Key Laboratory of Neonatal Diseases, Fudan University, Children’S Hospital of Fudan University, Shanghai, 201102 China; 6 Department of Health Research Methods, Evidence, and Impact, McMaster University, Hamilton, ON Canada; 7 Department of Neonatology, Children’S Hospital of Fudan University, 399 Wanyuan Road, Shanghai, 201102 China; 8 Maternal-Infants Care Research Centre, Mount Sinai Hospital, Toronto, ON M5G 1X5 Canada; 9 University of Toronto, Toronto, ON M5T 3M7 Canada

**Keywords:** Antenatal Corticosteroids, Neonatal Outcome, Very Preterm Infant, Hypertensive Disorders of Pregnancy

## Abstract

**Background:**

The relationship between antenatal corticosteroids (ACS) and preterm infants born to mothers with hypertensive disorders of pregnancy (HDP) remains a subject of debate. To evaluate whether the use of ACS before delivery was associated with neonatal outcomes in very preterm infants born to mothers with HDP.

**Methods:**

This multicenter cohort study enrolled all infants with gestational age at 24 to 31 week and admitted to tertiary NICUs of the Chinese Neonatal Network (CHNN) within 24 h of birth from 2019 to 2021. ACS administration was defined as at least one dose of dexamethasone or betamethasone before delivery. The primary outcome was surfactant and/ or invasive mechanical ventilation (IMV) within 72 h of life. Multivariable logistic regression analyses were performed to assess the association between ACS and neonatal outcomes.

**Results:**

Among the 4,582 study infants born to mothers with HDP, 3,806 (83.1%) were exposed to ACS. ACS treatment was significantly associated with lower risk of requirement of surfactant and/ or IMV within 72 h of life (adjusted Odds Ratio = 0.60, 95% confidence interval 0.49–0.74). ACS exposure was also independently associated with decreased mortality, surfactant use, IMV, combined surfactant and IMV use and moderate or severe bronchopulmonary dysplasia. The severity of maternal HDP did not appear to influence the correlation between ACS treatment and neonatal outcomes. Our analysis also indicated that a single complete course seemed to have the most significant protective effect.

**Conclusions:**

Our study reinforces the significant role of ACS in reducing severe respiratory morbidity and mortality in very preterm infants born to mothers with HDP.

**Supplementary Information:**

The online version contains supplementary material available at 10.1186/s13052-025-01909-9.

## Introduction

Preterm birth stands as a global challenge, significantly contributing to neonatal morbidity and mortality [1]. Antenatal corticosteroids (ACS) have been a cornerstone in the management of preterm delivery since the 1970s, shown to notably increase survival rates and reduce the incidence of major complications like respiratory distress syndrome (RDS), necrotizing enterocolitis (NEC), and intraventricular hemorrhage (IVH) in preterm infants [2]. Concurrently, hypertensive disorders of pregnancy (HDP) affect approximately 14.3% of all pregnancies worldwide, causing significant risks to both mother and infant [3].

While ACS has been widely used to infants born to mothers with HDP, the existing literature on its efficacy and safety in these specific cases remains limited and contradictory [4]. Previous studies, including several randomised clinical trials [5–8] and cohort studies [9–14] have been hindered by small sample sizes, inadequate control for confounders such as gestational age (GA), a focus on severe cases of maternal HDP, lack of subgroup analysis based on the severity of HDP and courses of ACS. These limitations have led to a lack of reliable clinical recommendations and raised concerns about the safety of ACS in HDP-affected pregnancies, particularly in the context of altered uterine perfusion and systemic changes like hypoxia and oxidative stress [15].

Therefore, our study aimed to investigate the association between ACS treatment and neonate outcomes among very preterm infants born to mothers with HDP. Utilizing data from the large nationwide cohort of the Chinese Neonatal Network (CHNN), this study seek to provide a more comprehensive understanding of ACS use in this high-risk group.

## Methods

### Study design and data source

This multicenter cohort study utilized data from the CHNN. CHNN database prospectively collected detailed clinical data of all infants born with a gestational age of < 32 weeks or a birth weight of < 1500g and admitted to participating neonatal intensive care units (NICUs) across China, aiming to offer a comprehensive overview of neonatal care practices and outcomes in China [16]. The CHNN database was initiated on January 1, 2019. In 2019, 57 NICUs contributed complete annual data to CHNN. The number of participating sites increased to 70 in 2020 and 79 in 2021. All NICUs included were tertiary-level and have the ability to provide comprehensive intensive neonatal care. Data collection was conducted by trained data abstractors at each site. Data quality was maintained by built-in error-checking mechanisms of the database, standardized data collection protocols and definitions, extensive data quality control process by the coordination center and periodic data audits [17].

### Ethics

The study was conducted following the Declaration of Helsinki and was approved by the Research Ethics Committee of the Children’s Hospital of Fudan University (Approval No. 2018-296>). A waiver of consent was universally granted due to the utilization of deidentified patient data.

### Study population

For this study, all infants admitted to participating hospitals within 24 hours after birth from 2019 to 2021 were included. Infants with severe congenital malformations, infants with antenatal steroids exposure because of other maternal diseases, unknown information of ACS usage or maternal HDP status, and infants born to mothers without HDP were excluded.

### Exposure

ACS administration was defined as at least one dose of dexamethasone or betamethasone before delivery. The time interval of ACS administration to birth was defined as the duration between the initial ACS dose and the time of delivery. Regarding ACS courses, a single complete course was identified as the administration of two doses of betamethasone or four doses of dexamethasone before delivery. A single partial course was characterized by the use of a single betamethasone dose or fewer than four dexamethasone doses prior to delivery. A repeat course was designated by the administration of more than one complete course of ACS.

### Outcomes

The most significant effect of ACS was accelerating lung maturation, so our primary outcome was the usage of surfactant and/ or IMV within 72 hours of life. The secondary outcomes included death, surfactant within 72 hours of life, IMV within 72 hours of life, combined surfactant and IMV use within 72 hours of life, bronchopulmonary dysplasia (BPD, defined as ventilation or oxygen dependency at 36 weeks’ postmenstrual age or discharge/transfer/death if before 36 weeks) [18], IVH (defined as ≥ grade 3 according to Papile’s criteria) [19], periventricular leukomalacia (PVL, defined as the presence of periventricular cysts on cranial ultrasound or MRI), NEC (defined as ≥ stage 2 according to Bell’s criteria) [20], sepsis (defined as positive blood or cerebrospinal fluid culture) and early-onset sepsis (EOS, defined as sepsis which occurs within 72 hours after birth).

### Definitions

HDP was diagnosed according to the 2015 Chinese Guideline on Hypertensive Disorders of Pregnancy [21]. This involves systolic blood pressure ≥140 mmHg and/or diastolic blood pressure ≥ 90 mmHg. HDP encompasses three categories: gestational hypertension, pre-existing hypertension, and hypertension but timing unknown. Gestational hypertension was identified if high blood pressure was first observed after 20 weeks of gestation, while pre-existing hypertension indicates that the mother had a history of hypertension prior to pregnancy. Hypertension but timing unknown applies to cases where the timing of hypertension onset could not be determined. For mothers with gestational hypertension or pre-existing hypertension, we further assessed whether they developed preeclampsia or eclampsia. Preeclampsia was characterized by proteinuria in addition to gestational hypertension. Eclampsia was defined as the occurrence of one or more convulsions in association with the preeclampsia syndrome. GA was determined using the hierarchy of best obstetric estimates based on prenatal ultrasound, menstrual history, obstetric examination, or all three. If the obstetric estimate was unavailable or differed from the postnatal estimate of gestation by more than two weeks, the GA was estimated using the Ballard Score [22]. Small for gestational age (SGA) was defined as birth weight <10th percentile for the gestational age according to the Chinese neonatal birth weight value [23]. A prenatal visit was defined as ≥ 1 pregnancy-related hospital visits during pregnancy. Chorioamnionitis included both clinical and pathological chorioamnionitis.

### Statistical analysis

Data were presented as frequency and percentage, mean with standard deviation (SD), or median with interquartile range (IQR) wherever appropriate. Differences in baseline characteristics and neonatal outcomes between ACS and no ACS groups were evaluated using χ^2^ test for categorical variables and Mann-Whitney U test for continuous variables.

Multilevel mixed-effect logistic regression models were used to evaluate the association between ACS and neonatal outcomes, adjusting for both patient-level confounders and hospitals to account for cluster effects. Patient-level confounders included GA, infant gender, SGA, multiple birth, gestational diabetes mellitus, premature rupture of membranes (PROM), prenatal visit, cesarean section, chorioamnionitis, inborn and magnesium sulfate. The selection of these confounders was guided by clinical experience, existing literature [9, 12, 24, 25], a directed acyclic graph (see online Suppl. 1) and were pre-determined prior to the study.

Subgroup analyses were also conducted to identify specific populations that might benefit most from ACS treatment. These analyses were stratified based on variables including maternal age, gestational diabetes mellitus, multiple births, GA, infant gender, and SGA. Additionally, the effect of ACS treatment may vary among different types of maternal HDP, so further subgroup analyses were conducted in infants born to mothers with preeclampsia/eclampsia and gestational hypertension. The interaction term of different subgroups and ACS treatment on the primary outcome was evaluated in the multivariable regression model mentioned above.

To explore the impact of diverse ACS usage patterns, including administration-to-birth intervals (≤7 days and >7 days) and different treatment courses (single partial course, single complete course, repeat courses), we applied similar statistical models, using the no-ACS group as the reference.

To ensure the robustness of the results, we conduced a sensitivity analysis using propensity score matching (PSM). This analysis used caliper matching (caliper=0.10) and included the same patient-level variables previously mentioned in the logistic regression model to calculated propensity score.

Data was analyzed using SAS 9.4 (SAS Institute Inc., Cary, NC, USA) and GraphPad Prism7 (GraphPad Software Inc.). Differences were considered statistically significant with a two-tailed *p*-value < 0.05.

## Results

### Study population and population characteristics

**Fig. 1:**
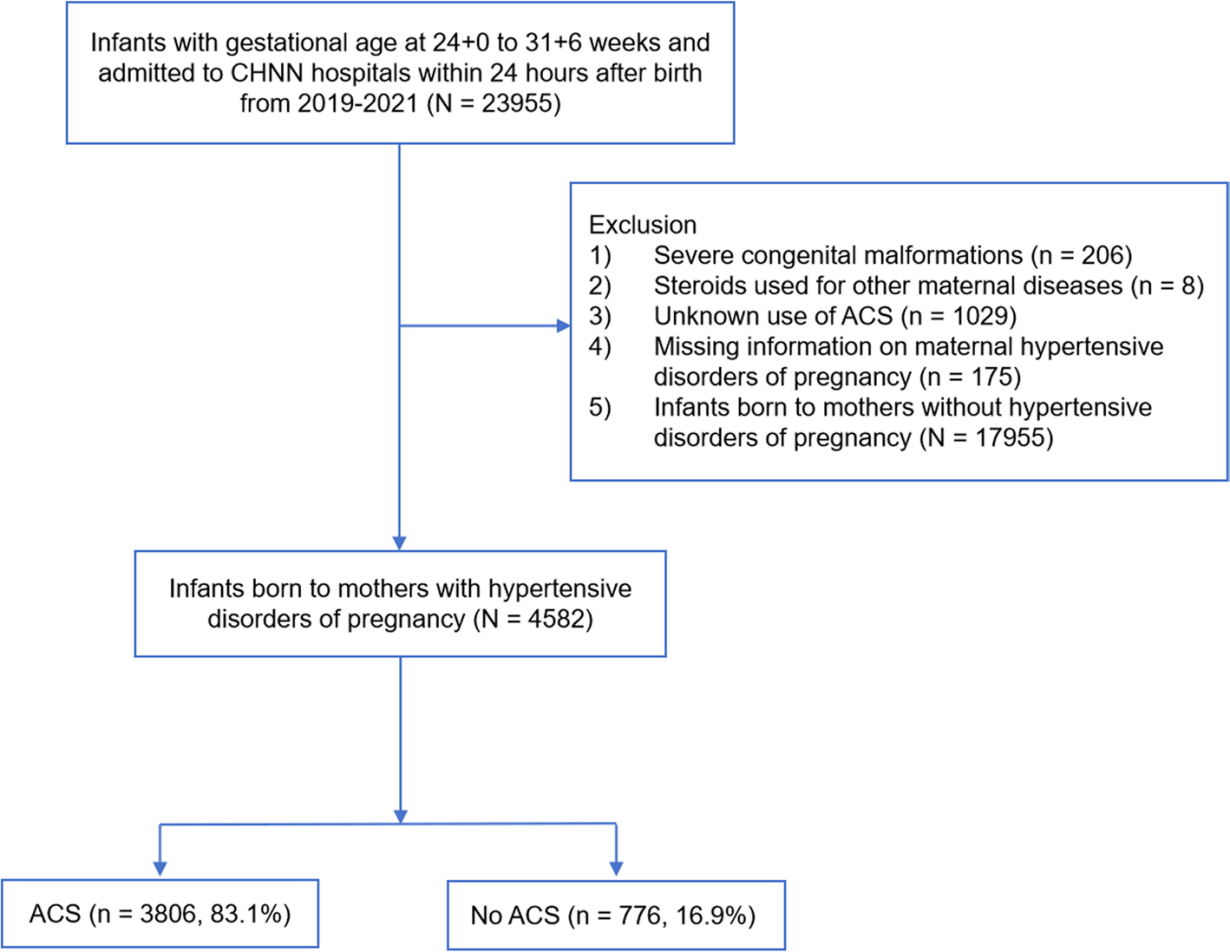
Flowchart of study population. ACS, antenatal corticosteroids

From 2019 to 2021, a total of 23,566 infants with gestational age at 24 to 31 weeks were admitted to CHNN NICUs within 24 hours after birth. Of these 4,582 infants born to mothers with HDP were included in this study. Among this cohort, 3,806 (83.1%) infants were exposed to ACS, while 776 (16.9%) infants were not (see Fig. 1).

For maternal characteristics, the ACS group showed significantly higher rates of cesarean section, PROM, antenatal antibiotics, magnesium sulfate, and chorioamnionitis compared to no ACS group (see Table 1). Preeclampsia/eclampsia was more prevalent the ACS group. For infant characteristics, the median gestational age were 30.3 weeks (IQR 29.1–31.1) in the ACS group and 30.0 weeks (IQR 29.0–31.0) in the no ACS group. The ACS group were more likely to be female and inborn, and less likely to have low Apgar scores at 5 minutes. Among infants with ACS exposure, 77.8% were born within seven days after the first ACS dose, and 59.9% received a single complete course of ACS (see Table 1).

**Table 1:** Maternal and infant characteristics of very preterm infants born to mothers with hypertensive disorders of pregnancy by antenatal corticosteroids use

	ACS (*n* = 3806)	No ACS (*n* = 776)	*P*-value
**Maternal characteristics**			
Maternal age, years,median (IQR)	32.0 (29.0–35.0)	31.5 (28.0–36.0)	0.204
≥ 35y, n (%)	1191 (31.3)	244 (31.4)	0.934
< 35y, n (%)	2615 (68.7)	532 (68.6)	
Primigravida, n/N (%)	1997/3788 (52.7)	428/770 (55.6)	0.146
Prenatal visit, n/N (%)	3688/3714 (99.3)	721/731 (98.6)	0.066
Cesarean section, n/N (%)	3501/3802 (92.1)	678/772 (87.8)	0.0001
PROM (≥ 24 h), n/N (%)	195/3727 (5.2)	20/728 (2.8)	0.015
Antenatal antibiotics, n/N (%)	1182/3567 (33.1)	178/705 (25.3)	< 0.0001
Magnesium sulfate, n/N (%)	2833/3587 (79.0)	353/710 (49.7)	< 0.0001
Pathological chorioamnionitis, n/N (%)	193/3031 (6.4)	25/597 (4.2)	0.041
Suspected Chorioamnionitis, n/N (%)	108/3130 (3.5)	12/624 (1.9)	0.048
Gestational diabetes mellitus, n/N (%)	747/3796 (19.7)	149/771 (19.3)	0.822
Type of hypertension			
Gestational hypertension, n (%)	3299 (86.7)	669 (86.2)	0.728
Pre-existing hypertension, n (%)	493 (12.9)	99 (12.8)	0.882
Hypertension but timing unknown, n (%)	14 (0.4)	8 (1.0)	0.015
Preeclampsia/eclampsia, n/N (%)	2967/3753 (79.1)	532/756 (70.4)	< 0.0001
Hypertensive disorders of pregnancy as primary reason for preterm birth, n (%)	2774 (72.9)	540 (69.6)	0.061
**Infant characteristics**			
Male, n/N (%)	1904/3802 (50.1)	422/775 (54.5)	0.027
Gestational age, weeks, median (IQR)	30.3 (29.1–31.1)	30.0 (29.0–31.0)	0.0009
< 28 weeks, n (%)	306 (8.0)	75 (9.7)	0.135
≥ 28 weeks, n (%)	3500 (92.0)	701 (90.3)	
Birth weight, grams, median (IQR)	1180 (1000–1370)	1155 (980–1360)	0.083
Small for gestational age, n (%)	935 (24.6)	190 (24.5)	0.962
Multiple birth, n (%)	690 (18.1)	127 (16.4)	0.242
Inborn, n (%)	3173 (83.4)	595 (76.7)	< 0.0001
Apgar 5 min ≤ 3, n/N (%)	28/3701 (0.8)	22/723 (3.0)	< 0.0001
**Uses of ACS**			
ACS administration-to-birth interval (n = 2616), n (%)			
≤ 7d	2036 (77.8)	NA	
> 7d	580 (22.2)	NA	
ACS courses (*n* = 3629), n (%)			
Single complete course	2172 (59.9)	NA	
Single partial course	946 (26.1)	NA	
Repeat courses	458 (12.6)	NA	

*IQR* interquartile range, *n/N* number and total number, *PROM* premature rupture of membrane, *ACS* antenatal corticosteroids, *NA* not available

### Neonatal outcomes

A lower proportion of infants in the ACS group (67.8%, 2,579/3,806) required surfactant administration and/or IMV within the first 72 hours of life compared to those in the no ACS group (76.7%, 595/776) (*P* < 0.0001, see Table 2). Additionally, the mortality rate was lower in the ACS group (9.1%) compared to the no ACS group (14.7%, *P* < 0.0001). The ACS group also exhibited reduced rates of surfactant use, IMV, combined surfactant and IMV use, and moderate or severe BPD. However, the incidences of IVH, PVL, NEC, sepsis, and EOS did not show significant differences between the two groups (see Table 2).

**Table 2:** Association between antenatal corticosteroids and neonatal outcomes among very preterm infants born to mothers with hypertensive disorders of pregnancy

	ACS (*n* = 3806) N/(%)	No ACS (*n* = 776) N/(%)	*P*-value	Crude OR (95% CI)	aOR^a^ (95% CI)
**Primary outcome**					
Surfactant and/or IMV within 72 h of life	2579 (67.8)	595 (76.7)	< 0.0001	0.61 (0.50–0.74)	0.60 (0.49–0.74)
**Secondary outcomes**					
Death	347 (9.1)	114 (14.7)	< 0.0001	0.53 (0.42–0.68)	0.54 (0.42–0.70)
Surfactant within 72 h of life	2335 (61.4)	509 (65.6)	0.026	0.77 (0.64–0.92)	0.77 (0.64–0.93)
IMV within 72 h of life	1450 (38.1)	402 (51.8)	< 0.0001	0.58 (0.49–0.69)	0.59 (0.49–0.71)
Combined surfactant and IMV use within 72 h of life	1206 (31.7)	316 (40.7)	< 0.0001	0.66 (0.55–0.78)	0.69 (0.57–0.82)
Moderate or severe BPD	1402/3787 (37.0)	374/771 (48.5)	< 0.0001	0.61 (0.52–0.72)	0.61 (0.51–0.73)
IVH (Grade III or IV)	184/3457 (5.3)	45/675 (6.7)	0.163	0.75 (0.52–1.08)	0.78 (0.53–1.14)
PVL	96/3441 (2.8)	26/673 (3.9)	0.133	0.76 (0.46–1.24)	0.86 (0.51–1.45)
NEC (Stage II or above)	183 (4.8)	34 (4.4)	0.610	1.10 (0.73–1.66)	1.06 (0.70–1.62)
Early-onset sepsis	29 (0.8)	7 (0.9)	0.687	0.69 (0.30–1.60)	0.58 (0.24–1.38)
Sepsis	335 (8.8)	73 (9.4)	0.590	0.92 (0.69–1.22)	0.95 (0.70–1.27)

aAdjusted for GA, infant gender, SGA, multiple birth, gestational diabetes mellitus, premature rupture of membranes, prenatal visit, cesarean section, chorioamnionitis, inborn, magnesium sulfate and hospital site

*IMV* invasive mechanical ventilation, *BPD* bronchopulmonary dysplasia, *IVH* intraventricular haemorrhage, *PVL* periventricular leukomalacia, *NEC* necrotizing enterocolitis, *ACS* antenatal corticosteroids

After adjustment, ACS exposure was associated with a decreased risk of surfactant and/or IMV within 72 hours of life (aOR 0.60, 95% CI 0.49–0.74) (see Table 2). ACS was also independently associated with reduced risk of death, surfactant use, IMV, combined surfactant and IMV use, and moderate or severe BPD.

### Subgroup analyses

**Fig. 2:**
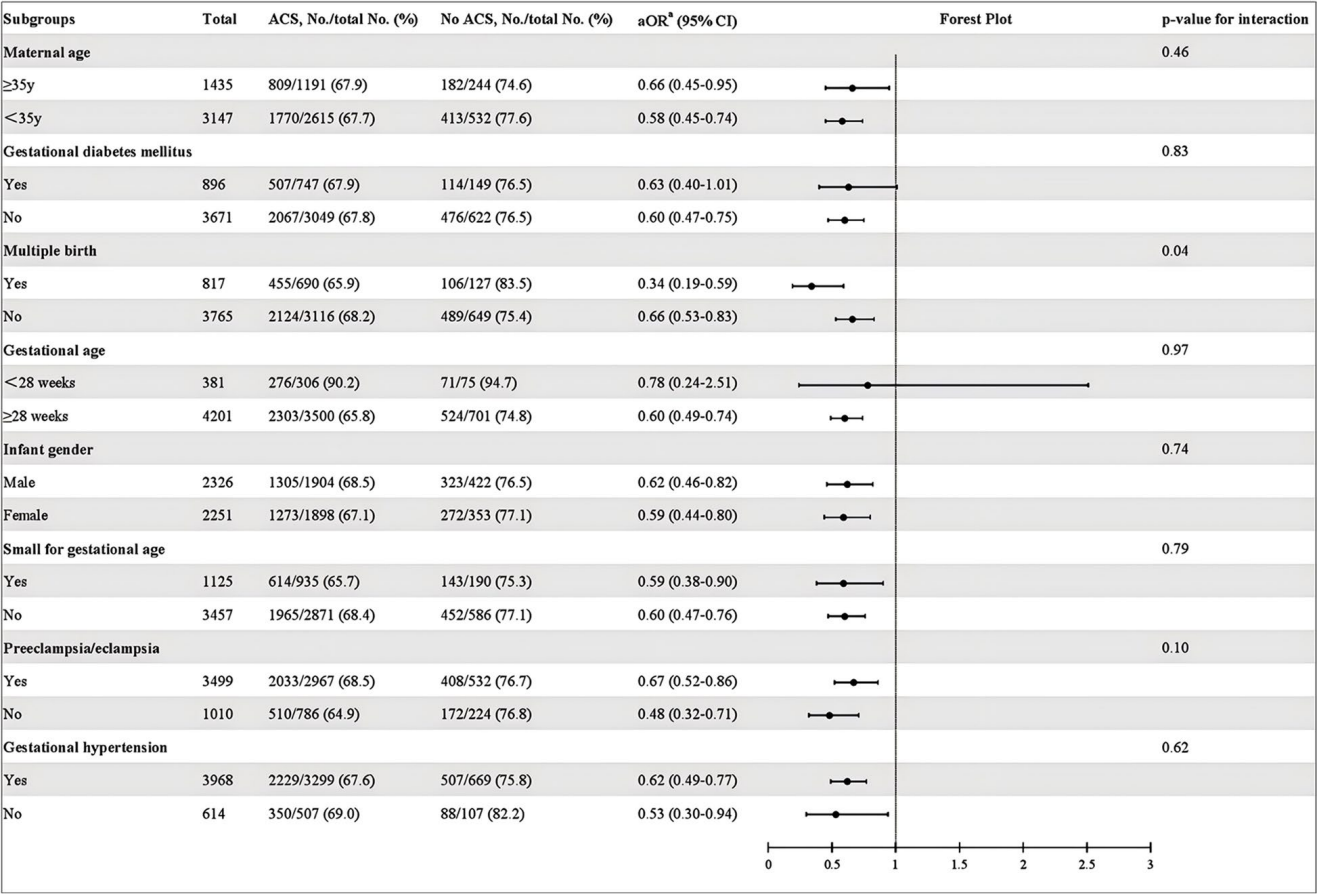
Subgroup analysis of the association between antenatal corticosteroids and surfactant and/or IMV within 72 h of life among infants born to mothers with hypertensive disorders of pregnancy. ACS, antenatal corticosteroids; No./total No., number and total number; OR, odds ratio; CI, confidence interval. ^a^Adjusted for GA, infant gender, SGA, multiple birth, gestational diabetes mellitus, premature rupture of membranes, prenatal visit, cesarean section, chorioamnionitis, inborn, magnesium sulfate and hospital site. When compared in gestational diabetes mellitus, multiple birth, gestational age infant gender and SGA groups, other confounders were adjusted

Subgroup analyses based on infant characteristics demonstrated a consistently lower risk of surfactant and/ or IMV use among infants with ACS exposure, with the exception of infants <28 weeks (see Fig. 2).

### Diverse ACS usage patterns

**Fig. 3:**
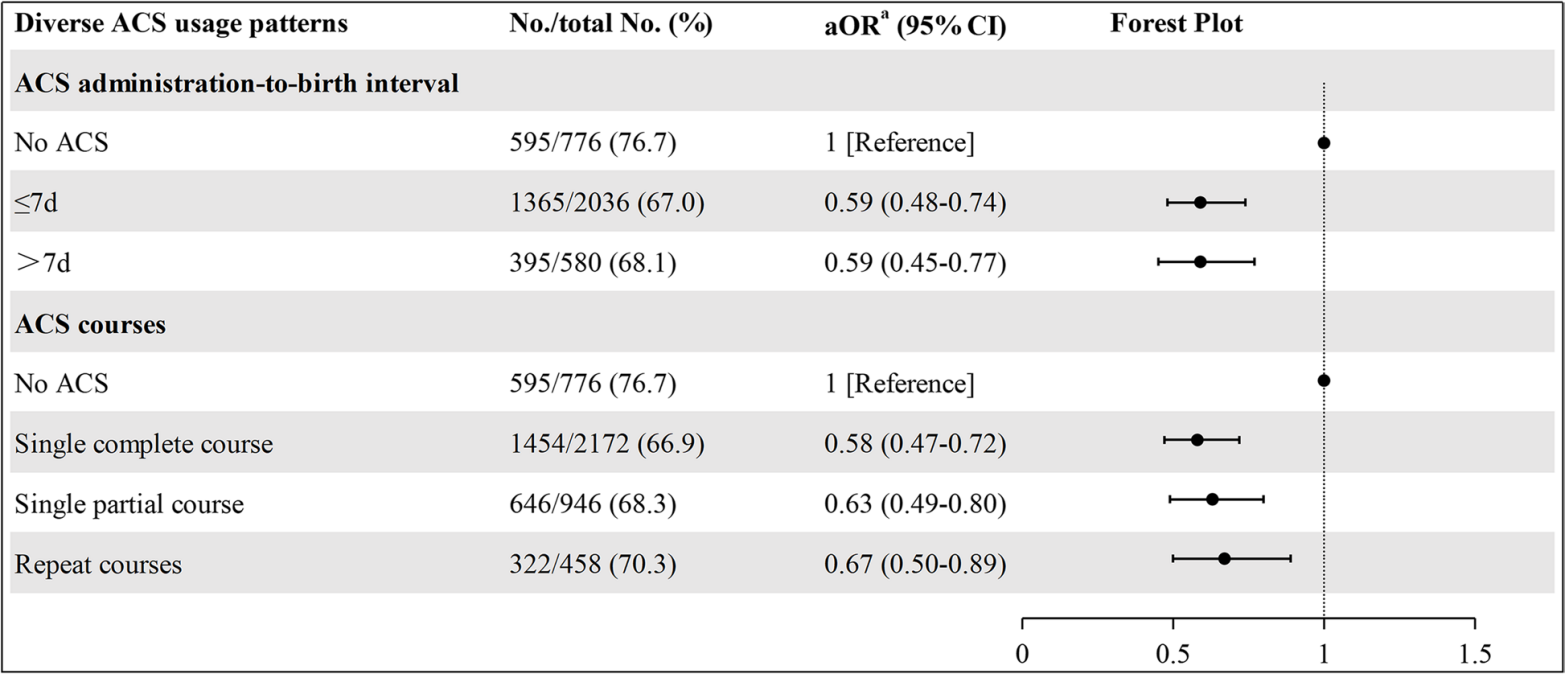
Subgroup analysis of the association between antenatal corticosteroids and surfactant and/or IMV within 72 h of life among infants born to mothers with hypertensive disorders of pregnancy (according to different uses of antenatal corticosteroids). ACS, antenatal corticosteroids; No./total No., number and total number; OR, odds ratio; CI, confidence interval. ^a^Adjusted for GA, infant gender, SGA, multiple birth, gestational diabetes mellitus, premature rupture of membranes, prenatal visit, cesarean section, chorioamnionitis, inborn, magnesium sulfate and hospital site

Data on ACS administration-to-birth intervals and treatment courses were available for 3,392 and 4,352 infants, respectively. Compared to infants without ACS exposure, ACS administered ≤7 days or >7 days before delivery demonstrated similar protective effects for the primary outcome (aOR 0.59, 95% CI 0.48–0.74; aOR 0.59, 95% CI 0.45–0.77). Additionally, different ACS courses—including single complete courses, single partial courses, and repeat courses—were all associated with beneficial effects when compared to the no-ACS group (aOR 0.58, 95% CI 0.47–0.72; aOR 0.63, 95% CI 0.49–0.80; aOR 0.67, 95% CI 0.50–0.89, respectively) (see Fig. 3).

### Sensitive analyses

In the sensitivity analysis using PSM, 646 infants from the no ACS group were successfully matched to 646 infants in the ACS group. In these matched cohorts, infants with ACS exposure had lower risk of surfactant and/ or IMV use compared to infants without ACS exposure (OR 0.67, 95% CI 0.52–0.87) (see online Suppl. 2).

## Discussion

Our study, using the largest concurrent multicenter cohort in China, confirmed that ACS was associated with significantly reduced respiratory morbidity and lower mortality in very preterm infants born to mothers with HDP. The severity of maternal HDP did not appear to influence the correlation between ACS treatment and neonatal outcomes. Our analysis also indicated that a single complete course seemed to have the most significant protective effect.

The beneficial impact of ACS exposure among very preterm infants whose mothers had HDP was evident with a 40% reduction in the need for surfactant and/or IMV in our study. This finding was consistent with data from various cohorts, including a national population-based cohort from Japan [12], a provincial population-based cohort from Canada [9], a cohort from USA [14], and findings from two trials [7, 8]. Our study reinforces these observations through thorough adjustments, multiple subgroup analyses, and sensitivity analyses, lending strong support to the current practice of prescribing ACS to pregnant women with HDP at risk of preterm birth. It is also noteworthy that the mothers in the ACS group exhibited higher rates of complications such as PROM, chorioamnionitis, and preeclampsia/eclampsia. This indicates a more complex prenatal condition in this group. Despite these challenges, infants exposed to ACS experienced less severe respiratory diseases. While on the other hand, we should also notice the increased likelihood of cesarean delivery and magnesium sulfate administration in the ACS group. This reflects a more controlled and attentive perinatal care approach in this group, which may amplify the effect of ACS.

However, some RCTs [5, 6] and cohort studies [10, 13] have reported no improvement in outcomes with ACS treatment. There might be several reasons for the conflicting results. First, these studies were based on relatively small sample sizes [5, 6, 10], which might have limited their ability to detect a positive effect. Second, some studies enrolled more mature preterm infants (<35 weeks [13] or <37weeks [5]), and these infants were less likely to have severe respiratory diseases. Pooling data across gestational ages may mask the potential effect of ACS treatment on RDS for the more immature population. Third, these studies enrolled the most severe cases of maternal HDP [5, 10, 13]. Our study did not demonstrate a significant beneficial effect of ACS treatment among infants <28 weeks, despite this group being the most likely to benefit from such intervention. This finding may be partially explained by several factors. First, the relatively small sample size of infants <28 weeks in our cohort limited the statistical power to detect potential benefits. Second, these infants had a high rate of surfactant administration and/or IMV within 72 hours of life, reflecting the intensive respiratory support commonly provided in Chinese NICUs. Such high intervention rates may have masked the potential effects of ACS treatment by reducing observable differences in outcomes. Third, variations in perinatal care practices, including the timing and completeness of ACS administration, could have introduced variability, further influencing the results. These findings highlight the need for larger, more focused studies to evaluate the impact of ACS specifically in infants <28 weeks.

We observed that ACS treatment was also associated with a reduced risk of mortality. This correlation aligns with findings from previous studies [7, 12]. Additionally, in our study, ACS treatment was linked to a decreased risk of BPD, in contrast to a Japanese study [12]. The discrepancy could be attributed to differences in the study populations, such as a higher proportion of extremely premature infants in the Japanese cohort, and variations in the definition of BPD. Nevertheless, the impact of ACS treatment on BPD warrants further investigation in future studies.

While our study revealed that ACS treatment in very preterm infants was linked to a reduced requirement of surfactant and/ IMV use, irrespective of different patterns of ACS usage, the beneficial effect seemed most significant among infants with a single complete course. This finding was similar with previous studies in which women who received multiple courses or complete course of ACS did not improve preterm-birth outcomes over women receiving placebo or partial course of ACS, and these results might suggest a dose-dependent effect of ACS [26, 27].

Our study has several strengths. First, the large sample size allowed us to perform extensive adjustment and subgroup analyses which contributed to the robustness of our results. The comprehensive data collection on timing and course of ACS administration, along with the severity of maternal HDP, allowed for analyses of the effects of different ACS treatment patterns and the impact of maternal HDP severity.

Our study also has several limitations. First, we lacked specific details on the timing of hypertension onset and blood pressure control. To mitigate, we incorporated the presence or absence of preeclampsia/eclampsia as an indicator of hypertension severity. Second, the Ballard score was used to estimate GA when the obstetric estimate was unavailable or when there was a discrepancy of more than two weeks between the obstetric and postnatal estimates. However, it is important to note that the Ballard score, despite being widely utilized, tends to overestimate GA and exhibits wide margins of error, particularly in infants born SGA [28]. Third, we did not have information on fetal death or other maternal complications, which could have provided a more comprehensive understanding of the effects of ACS. Fourth, we chose the usage of surfactant and/or IMV as an indicator of acute respiratory severity based on clinical knowledge and previous literature [29], while treatment selections might vary among hospitals and clinicians. We adjusted for various hospital sites to minimize differences, but residue bias may exist. Fifth, it should be noted that the overall rate of surfactant and/or IMV use was high in our population, particularly among infants <28 weeks. Therefore, the results may not be generalizable to regions with differing practices. Specifically, we lack detailed information on the type, dose, and retreatment protocols for surfactant, as well as the methods of respiratory support employed (noninvasive vs. invasive ventilation). These factors could have influenced the association between ACS and respiratory outcomes in this population and may explain the lack of observed benefit in our study.

## Conclusions

In summary, our study reinforces the significant role of ACS in reducing severe respiratory morbidity and mortality in very preterm infants born to mothers with HDP. Further follow-up studies are needed to deepen our understanding and improve the management of such pregnancies. Additional research is required to evaluate the association between ACS and neonatal outcomes in infants <28 weeks.

## Supplementary Information

Supplementary Material 1

## Data Availability

All relevant raw data can be freely available to any researcher wishing to use them for non-commercial purposes from the corresponding author.

## Abbreviations

ACS: Antenatal corticosteroids
HDP: Hypertensive disorders of pregnancy
IMV: Invasive mechanical ventilation
BPD: Bronchopulmonary dysplasia
IVH: Intraventricular haemorrhage
PVL: Periventricular leukomalacia
NEC: Necrotizing enterocolitis
GA: Gestational age
SGA: Small for gestational age
PROM: Premature rupture of membrane
