# Supplementary material for: Association between antenatal corticosteroids and neonatal outcomes among very preterm infants born to mothers with hypertensive disorders of pregnancy: a multicenter cohort study
Source: Ital J Pediatr. 2025 Mar 13;51:75. doi: 10.1186/s13052-025-01909-9 (PMC11905651; doi:10.1186/s13052-025-01909-9)
Supplement: Supplementary file 1 — Supplementary Material 1 [file 13052_2025_1909_MOESM1_ESM.docx]

Online Suppl. 1. Directed acyclic graph map.


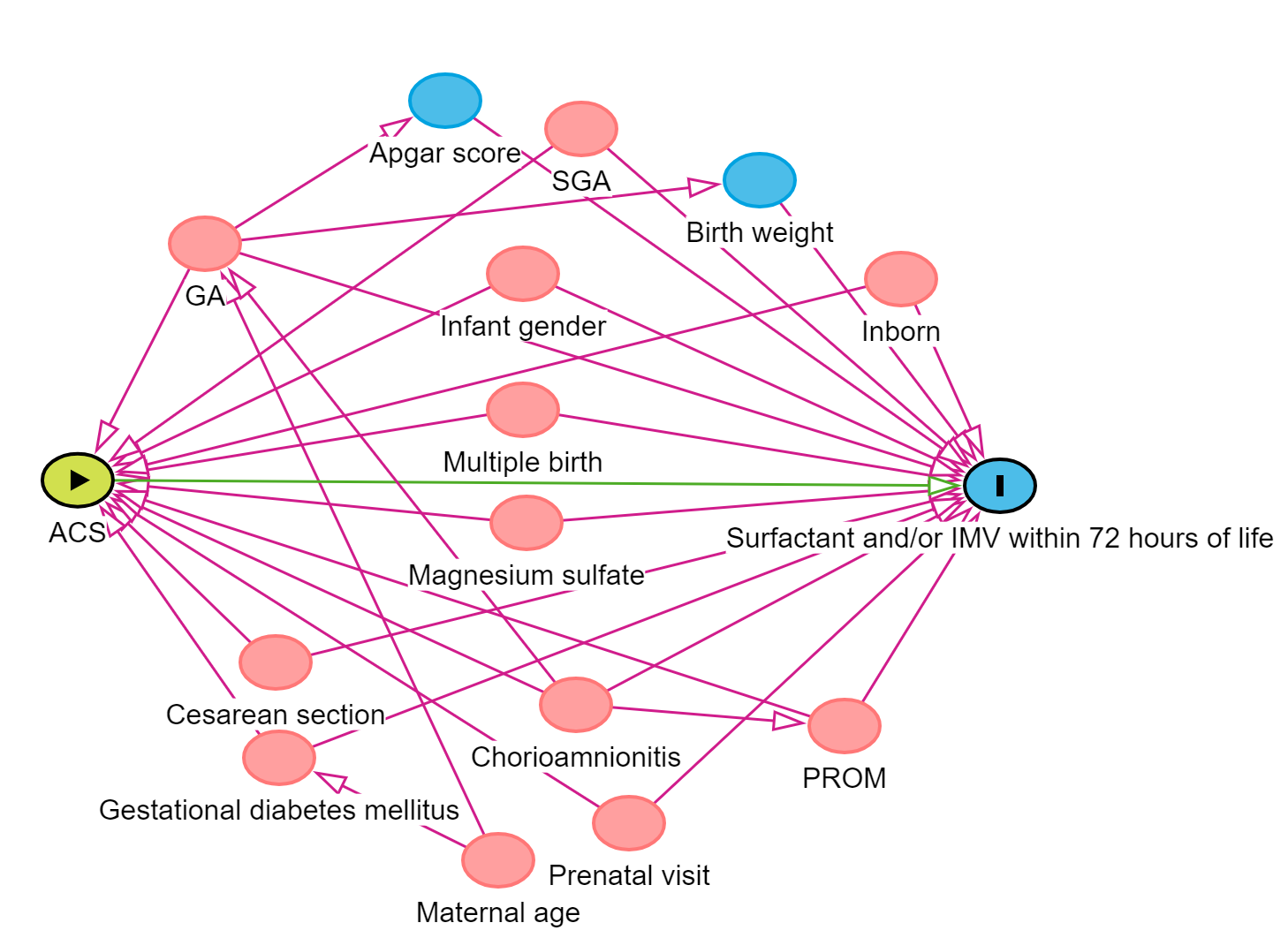


Online Suppl. 2. Sensitivity analysis-propensity score matched analysis.

Infants born to mothers with hypertensive disorders of pregnancy (N = 4582, 20.3%)

Propensity score match

ACS (n = 3806, 83.1%)

No ACS (n = 776, 16.9%)

130 not included in propensity score matched analysis

3160 not included in propensity score matched analysis

646 included in propensity score matched analysis^a^

646 included in propensity score matched analysis

Online Suppl. 2.1. Flowchart of study population in the propensity score matched sample.

ACS, antenatal corticosteroids. ^a^Covariables included in propensity score model were GA, infant gender, SGA, multiple birth, gestational diabetes mellitus, premature rupture of membranes, prenatal visit, cesarean section, inborn and magnesium sulfate.

Online Suppl. 2.2. Maternal and infant characteristics of very preterm infants born to mothers with hypertensive disorders of pregnancy by antenatal corticosteroids use in the propensity score matched sample.

|  | ACS (n = 646) | No ACS (n = 646) | P-value |
| --- | --- | --- | --- |
| **Maternal characteristics** | | | |
| Maternal age, years, median (IQR) | 32.0 (29.0-35.0) | 32.0 (29.0-36.0) | 0.826 |
| ≥35y, n (%) | 184 (28.5) | 205 (31.7) | 0.203 |
| ＜35y, n (%) | 462 (71.5) | 441 (68.3) |  |
| Primigravida, n/N (%) | 316/642 (49.2) | 360/642 (56.1) | 0.014 |
| Prenatal visit, n (%) | 645 (99.9) | 641 (99.2) | 0.102 |
| Cesarean section, n (%) | 566 (87.6) | 565 (87.5) | 0.933 |
| PROM (≥24h), n (%) | 19 (2.9) | 19/ (2.9) | 0.940 |
| Antenatal antibiotics, n/N (%) | 210/623 (33.7) | 170/618 (27.5) | 0.018 |
| Magnesium sulfate, n (%) | 332 (51.4) | 332 (51.4) | 1 |
| Pathological chorioamnionitis, n/N (%) | 34/521 (6.5) | 24/533 (4.5) | 0.150 |
| Suspected Chorioamnionitis, n/N (%) | 14/530 (2.6) | 11/558 (2.0) | 0.461 |
| Gestational diabetes mellitus, n (%) | 123 (19.0) | 135 (20.9) | 0.404 |
| Preeclampsia/eclampsia, n/N (%) | 470/639 (73.6) | 442/633 (69.8) | 0.140 |
| Gestational hypertension, n (%) | 568 (87.9) | 552 (85.5) | 0.190 |
| Hypertensive disorders of pregnancy as primary reason for preterm birth, n (%) | 468 (72.5) | 452 (70.0) | 0.326 |
| **Infant characteristics** | | | |
| Male, n (%) | 357 (55.3) | 344 53.3 | 0.468 |
| Gestational age, weeks, median (IQR) | 30.3 (29.0-31.1) | 30.1 (29.0-31.0) | 0.053 |
| ＜28 weeks, n (%) | 62 (9.2) | 67 (10.4) | 0.643 |
| ≥28 weeks, n (%) | 584 (90.4) | 579 (89.6) |  |
| Birth weight, grams, median (IQR) | 1192.5 (1000-1410) | 1160 (980-1366) | 0.077 |
| Small for gestational age, n (%) | 142 (22.0) | 143 (22.1) | 0.947 |
| Multiple birth, n (%) | 114 (17.7) | 112 (17.3) | 0.884 |
| Inborn, n (%) | 568 (87.9) | 563 (87.2) | 0.674 |
| Apgar 5min ≤3, n (%) | 3/638 (0.5) | 15/615 (2.4) | 0.0034 |
| **Uses of ACS** | | | |
| **ACS administration-to-birth interval (n=624), n (%)** | | | |
| ≤7d | 333 (53.4) | NA |  |
| ＞7d | 109 (17.5) | NA |  |
| **ACS courses (n=615), n (%)** | | | |
| Single complete course | 374 (60.8) | NA |  |
| Single partial course | 167 (27.2) | NA |  |
| Repeat courses | 69 (11.2) | NA |  |
| IQR, interquartile range; n/N, number and total number; PROM, premature rupture of membrane; ACS, antenatal corticosteroids; NA, not available. | | | |

Online Suppl. 2.3. Association between antenatal corticosteroids and neonatal outcomes among very preterm infants born to mothers with hypertensive disorders of pregnancy in the propensity score matched sample.

|  | ACS (n = 646)  N/(%) | No ACS (n = 646)  N/(%) | P-value | OR (95% CI) |
| --- | --- | --- | --- | --- |
| **Primary outcome** | | | | |
| Surfactant and/or IMV within 72 hours of life | 455 (70.4) | 502 (77.7) | 0.0028 | 0.67 (0.52-0.87) |
| **Secondary outcomes** | | | | |
| Death | 58 (9.0) | 102 (15.8) | 0.0002 | 0.48 (0.33-0.70) |
| Surfactant within 72 hours of life | 420 (65.0) | 440 (68.1) | 0.238 | 0.86 (0.68-1.10) |
| IMV within 72 hours of life | 231 (35.8) | 328 (50.8) | ＜0.0001 | 0.51 (0.40-0.65) |
| Combined surfactant and IMV use within 72 hours of life | 196 (30.3) | 266 (41.2) | ＜0.0001 | 0.60 (0.47-0.76) |
| Moderate or severe BPD | 225/644 (34.9) | 308/645 (47.8) | ＜0.0001 | 0.56 (0.44-0.71) |
| IVH (Grade III or IV) | 44/583 (7.6) | 39/554 (7.0) | 0.742 | 1.00 (0.61-1.63) |
| PVL | 18/577 (3.1) | 20/553 (3.6) | 0.643 | 0.68 (0.34-1.39) |
| NEC (Stage II or above) | 29 (4.5) | 28 (4.3) | 0.892 | 1.04 (0.61-1.78) |
| Early-onset sepsis | 6 (0.9) | 7 (1.1) | 0.780 | 0.86 (0.29-2.55) |
| Sepsis | 57 (8.8) | 64 (9.9) | 0.504 | 0.88 (0.60-1.28) |
| IMV, invasive mechanical ventilation; BPD, bronchopulmonary dysplasia; IVH, intraventricular haemorrhage; PVL, periventricular leukomalacia; NEC, necrotizing enterocolitis; n/N, number and total number; OR, odds ratio; CI, confidence interval. | | | | |
